# Expansion of cytotoxic natural killer cells using irradiated autologous peripheral blood mononuclear cells and anti-CD16 antibody

**DOI:** 10.1038/s41598-017-09259-1

**Published:** 2017-09-11

**Authors:** Hong-Rae Lee, Cheol-Hun Son, Eun-Kyoung Koh, Jae-Ho Bae, Chi-Dug Kang, Kwangmo Yang, You-Soo Park

**Affiliations:** 1 Department of Research Center, Dongnam Institute of Radiological & Medical Sciences, Jwadong-gil 40, Jangan-eup, Gijang-gun, Busan, 46033 South Korea; 2 Department of Biochemistry, Pusan National University School of Medicine, Yangsan, 50612 South Korea

## Abstract

Natural killer (NK) cells are considered a promising strategy for cancer treatment. Various methods for large-scale NK cell expansion have been developed, but they should guarantee that no viable cells are mixed with the expanded NK cells because most methods involve cancer cells or genetically modified cells as feeder cells. We used an anti-CD16 monoclonal antibody (mAb) and irradiated autologous peripheral blood mononuclear cells (PBMCs) (IrAPs) to provide a suitable environment (activating receptor-ligand interactions) for the NK cell expansion. This method more potently expanded NK cells, and the final product was composed of highly purified NK cells with lesser T-cell contamination. The expanded NK cells showed greater upregulation of various activation receptors, CD107a, and secreted larger amounts of interferon gamma. IrAPs expressed NKG2D ligands and CD48, and coengagement of CD16 with NKG2D and 2B4 caused potent NK cell activation and proliferation. The expanded NK cells were cytotoxic toward various cancer cells *in vitro* and *in vivo*. Moreover, irradiation or a chemotherapeutic drug further enhanced this antitumor effect. Therefore, we developed an effective *in vitro* culture method for large-scale expansion of highly purified cytotoxic NK cells with potent antitumor activity using IrAPs instead of cancer cell-based feeder cells.

## Introduction

Natural killer (NK) cells constitute approximately 10–15% of the lymphocytes in humans and are usually defined as CD3^−^CD56^+^ cells^1^. The primary function of NK cells is immune surveillance of the body. They play an important role in early immune responses by removing viral infections and cancer without recognizing specific antigens^2–4^. In particular, they can effectively inhibit the growth of cancer stem-like cells as well as tumor growth and metastasis in the human body^5–7^. The effector function of NK cells is determined by the balance between activating and inhibitory receptor signals^8^. An NK cell activating signal is mediated by various NK cell receptors, including CD16 (Fcγ-receptor), natural killer group 2D (NKG2D), 2B4, and natural cytotoxicity receptors (NCRs; NKp30, NKp44, NKp46, and NKp80)^8, 9^. In contrast, an NK cell inhibitory signal mainly is mediated by killer cell immunoglobulinlike receptors (KIRs) and CD94/NKG2A, which recognize major histocompatibility complex (MHC) class I molecules on target cells. Thus, MHC class I-deficient cancer or transformed cells are highly sensitive to NK cells^8, 10^. Hence, NK cells are considered a promising therapeutic option for cancer treatment, and numerous clinical studies have been performed on various tumors^7, 11^.

NK cell activation is synergistically augmented by coengagement of other activating receptors such as NKG2D and 2B4^12, 13^. NKG2D is a key member of activating receptors present on the surface of NK cells and performs an important function in the elimination of target cells^14, 15^. NKG2D recognizes the MHC class I-related chain A and B (MICA/B) and UL-16-binding proteins (ULBPs), which are induced by various stressors, including heat shock, ionizing radiation, oxidative stress, and viral infection^16, 17^. These NKG2D ligands show various expression patterns in different target cells^17^.

2B4 (CD244) is one of the well-known NK cell-activating receptors. The ligand of 2B4, CD48, is broadly expressed on hematopoietic cells, including NK cells themselves. 2B4-CD48 interactions predominantly induce NK cell activation through recruiting the small adaptor SAP bound to the tyrosine kinase Fyn^12, 13^. Recently, it was reported that 2B4-mediated signaling is intimately involved in augmenting NK cell activation and proliferation both *in vitro* and *in vivo* ^18^.

NK cells express CD16 (FcγRIII), a low-affinity receptor for IgG; this receptor is responsible for antibody-dependent cellular cytotoxicity (ADCC). ADCC is one of the major factors for the efficacy of antibody-based cancer therapies^19^. Most CD56^dim^ NK cells show high-density expression of CD16 but CD56^bright^ NK cells lack the expression of CD16 or show low-density expression^1^. In particular, CD16 has a unique ability to induce NK cell activation without additional receptor signals^13^. Recently, it was reported that individual receptor-ligand interactions are not sufficient to induce efficient activation of resting NK cells^12, 13, 20^. Thus, combinations of NK cell-activating receptors, including CD16 can be a promising strategy to induce NK cell activation and expansion. Various studies have focused only on the activity of NK cells by coengagement of activating receptors and have not addressed the expansion of NK cells. Therefore, we tried to induce not only the activity of NK cells but also large-scale expansion of these cells by means of combinations of activating receptors.

Lately, it is believed that one of the key factors in the success of NK cell-based cancer immunotherapy is dependent on obtaining a sufficient number of highly cytotoxic NK cells. Numerous studies have explored *ex vivo* activation and expansion of NK cells from a variety of sources. NK cells can be generated from cord blood, bone marrow, embryonic stem cells, and peripheral blood^11, 21^. A variety of cytokines, such as interleukin (IL)-2, IL-12, IL-15, IL-18, and IL-21 or their combinations have been used to expand NK cells^22–24^, but these cytokines were not very effective. For NK cell activation and expansion, cancer cell lines^25^, genetically modified K562 cells (artificial antigen-presenting cells with membrane-bound MICA, 4-1BBL, membrane-bound IL-15 and IL-21)^26–28^, or Epstein–Barr virus-transformed lymphoblastoid cell lines^29^ have been used as feeder cells (irradiated). Even though these methods have made large-scale NK cell expansion possible, they used cancer cell-based feeder cells. Therefore, it is important to control their growth and to ensure that no viable feeder cells are mixed with the expanded NK cells.

In this study, we used irradiated autologous peripheral blood mononuclear cells (PBMCs) (IrAPs) instead of cancer cell-based feeder cells for large-scale expansion of highly purified cytotoxic NK cells. Radiation upregulates NKG2D ligands and CD48 (a 2B4 ligand) in human PBMCs. Nonetheless, irradiated autologous PBMCs alone did not induce efficient expansion of NK cell. To overcome thus problems, we used an anti-CD16 monoclonal antibody (mAb) for potent activation of resting NK cells and added IrAPs (upregulated NKG2D ligand and CD48) for providing a suitable environment (activating receptor-ligand interactions and soluble growth factors) for the NK cell expansion. These expanded NK cells showed potent cytotoxicity against various cancer cells *in vitro* and efficiently controlled cancer progression in SCID mouse models of human colon and lung cancer. Thus, the proposed method provides robust expansion of highly purified cytotoxic human NK cells for adoptive immunotherapy using IrAPs instead of cancer cell-based feeder cells, and results in potent antitumor activity both *in vitro* and *in vivo*.

## Results

### Irradiation induces T-cell inactivation and upregulates NKG2D ligands and CD48 in human PBMCs

**Figure 1:**
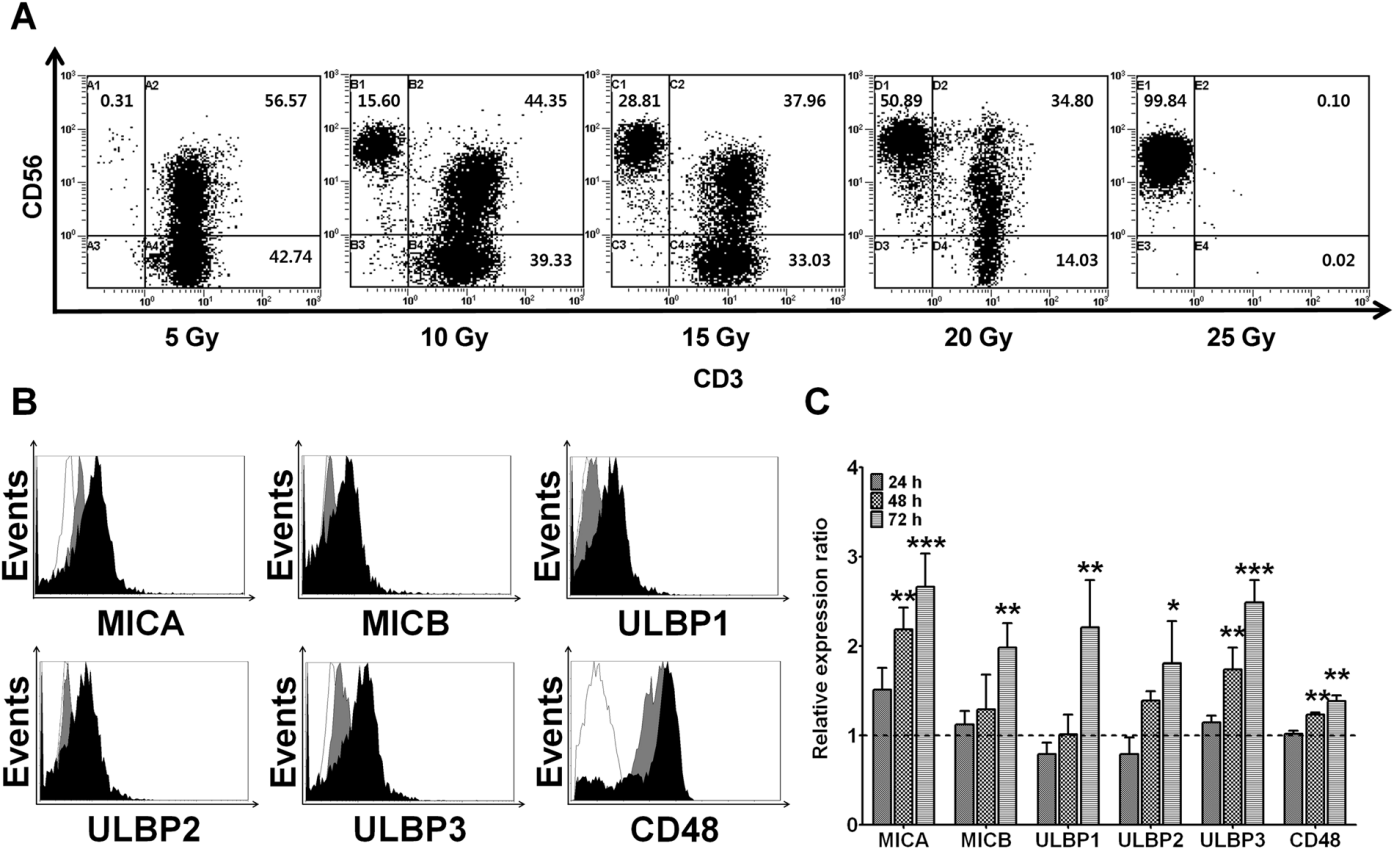
Radiation effectively induces T-cell inactivation and upregulates NKG2D ligands and CD48 in human PBMCs. The proportion of T cells and expression of NKG2D ligands and CD48 were measured by flow cytometry (FCM). (**A**) Results of phenotypic analysis by two-color FCM after 21 days. (**B**) Ratios of mean fluorescence intensities (MFIs) obtained from irradiated PBMCs and untreated control (fresh PBMCs). Relative expression ratios were calculated by dividing irradiated PBMC MFI by fresh PBMC MFI. (**C**) Representative FCM histograms (white: isotype control, gray: fresh PBMCs, black: 72 h after irradiation). All the experiments were conducted in triplicate for each donor (*n* = 5). The statistical significance was determined using paired Student’s *t* test. *P < 0.05, **P < 0.005, ***P < 0.0005 (*fresh PBMCs *versus* irradiated PBMCs).

To determine the optimal dose of radiation for T-cell inactivation, PBMCs were exposed to radiation doses of 5, 10, 15, 20, or 25 Gy. Then, the irradiated PBMCs were cocultured with resting NK cells for 21 days. The proportion of T cells was assessed by flow cytometry (Fig. 1A). T cells were clearly detectable during NK cell expansion after radiation doses of 5, 10, 15, and 20 Gy. Nevertheless, the radiation dose of 25 Gy induced effective inactivation of T cells during NK cell expansion. Therefore, we decided to use the 25-Gy irradiation, which can effectively induce inactivation of T cells. To test whether irradiation induces the expression of NKG2D ligands (MICA, MICB, and ULBP1-3) and CD48 (a 2B4 ligand) in human PBMCs, isolated PBMCs from donors were harvested 0, 24, 48, or 72 h after irradiation at 25 Gy. The analysis was based on flow cytometry, and cell surface expression levels were quantified using mean fluorescence intensities (MFIs; Fig. 1B,C). Relative expression ratios were calculated by dividing irradiated PBMCs’ MFI by fresh PBMCs’ MFI. The irradiated PBMCs expressed larger amounts of MICA, ULBP3, and CD48 compared to fresh PBMCs after 2 days, whereas MICB, ULBP1, and ULBP2 expression increased 3 days after the irradiation. Although PBMCs highly expressed CD48, this expression was further increased 2 days after the irradiation. These results indicate that the radiation dose of 25 Gy induces effective inactivation of T cells and upregulates NKG2D ligands and CD48 (2B4 ligand) in human PBMCs.

### A combination of the anti-CD16 (αCD16) mAb with irradiated autologous PBMCs (IrAPs) synergistically enhances expansion of NK cells

**Figure 2:**
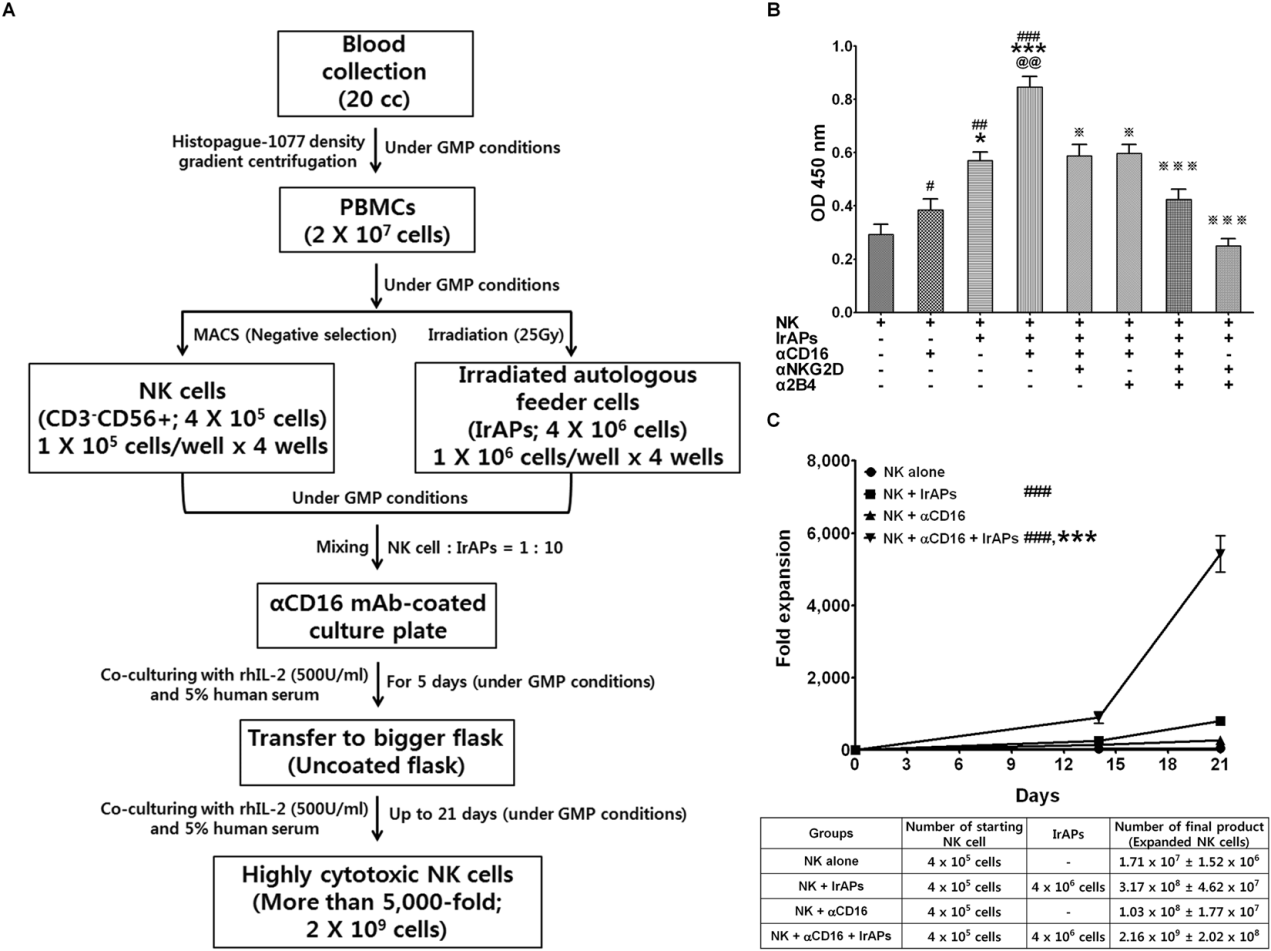
A combination of the anti-CD16 (αCD16) mAb with irradiated autologous PBMCs (IrAPs) strongly induces NK cell expansion. NK cells isolated from PBMCs were expanded under various culture conditions described in the Methods. (**A**) Schematic representation for expansion procedure of highly cytotoxic NK cell by the combination of the αCD16 mAb and IrAPs. (**B**) NK cell proliferation was analyzed using the EZ-Cytox Cell viability assay kit on day 5. (**C**) The fold expansion of NK cells was determined before (day 0) and after (days 14 and 21) NK cell expansion. The graphs show mean ± SD of five donors. The assay was conducted in triplicate for each donor. The statistical significance was determined by one-way ANOVA. ^#^ *P* < 0.05, ^##^ *P* < 0.005, ^###^ *P* < 0.0005 (^#^NK alone *versus* other groups). *P < 0.05, ***P < 0.0005 (*NK + αCD16 *versus* other groups). ^@@^ *P* < 0.005 (^@^NK + IrAPs *versus* NK + αCD16 + IrAPs). ^※^ *P* < 0.05, ^※※※^ *P* < 0.0005 (^※^NK + αCD16 + IrAPs *versus* other groups).

To examine the efficiency of NK cell expansion by a combination of the αCD16 mAb with IrAPs, resting NK cells from five donors were isolated and cultured *in vitro* with the αCD16 mAb and IrAPs under Good Manufacturing Practices (GMP) conditions (Fig. 2A). As shown in Fig. 2B, although irradiated autologous PBMCs strongly induced the initial proliferation of the resting NK cells, the proportion of expanded NK cells was more strongly increased by the combination of the αCD16 mAb with IrAPs. By contrast, the αCD16 mAb alone did not strongly induce the initial proliferation of NK cells as compared to the other groups. To confirm that NK cell proliferation is due to the synergistic combinations of activating receptors CD16, NKG2D, and 2B4, next, NKG2D-blocking and/or 2B4-blocking antibody-coated NK cells were cocultured with IrAPs. The blockade of receptor NKG2D or 2B4 significantly decreased NK cell expansion that is induced by the combination of the αCD16 mAb with IrAPs. In particular, the combined blockade more strongly inhibited NK cell expansion that is induced by the combination of the αCD16 mAb with IrAPs. These results indicate that NK cell expansion is induced by coactivation of receptors NKG2D and 2B4, and this effect was more strongly induced by synergistic combinations of receptors CD16, NKG2D, and 2B4. As shown in Fig. 2C, IL-2 alone (42.8 ± 3.8 fold) fails to expand NK cells, whereas NK cells stimulated with IrAPs were expanded 794 ± 115.6 fold and those stimulated with the αCD16 mAb were expanded 259.2 ± 44.4 fold. In particular, NK cells stimulated with the αCD16 mAb and IrAPs were expanded 5421.6 ± 505.4 fold. This interaction points to a synergistic effect of the αCD16 mAb and IrAPs. Thus, we demonstrated that a combination of the αCD16 mAb with IrAPs synergistically enhances the expansion of NK cells, and these receptor-ligand interactions are essential for the induction of robust expansion of NK cells.

### The combination of the αCD16 mAb with IrAPs increases expression of NK cell-activating receptors

**Figure 3:**
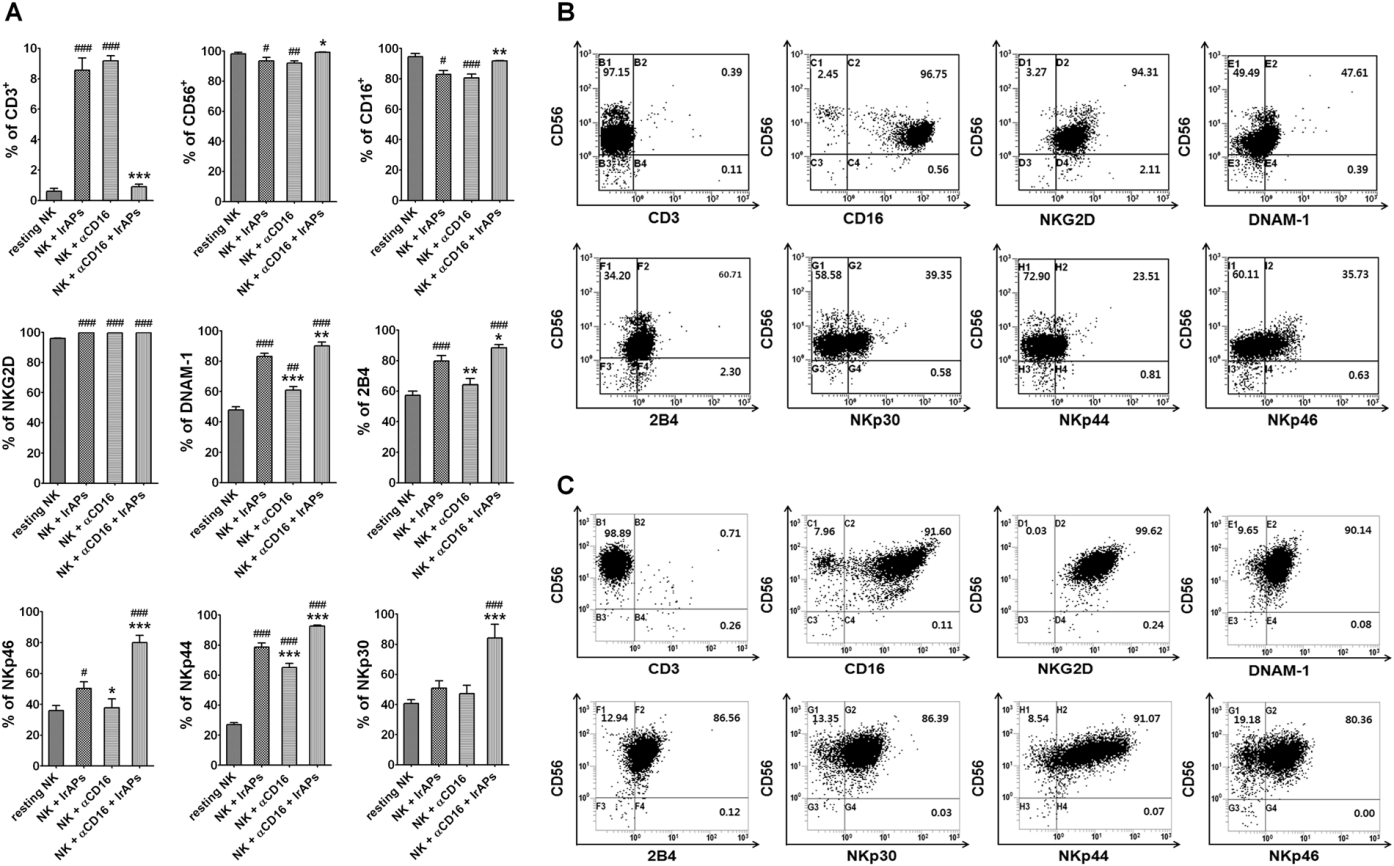
NK cell-activating receptors are upregulated by the combination of the αCD16 mAb with IrAPs. NK cell activation receptors were analyzed by flow cytometry (FCM). (**A**) Comparison of expression levels of NK cell receptors between days 0 and 21. (**B**,**C**) A representative FCM dot plot of resting NK cells (**B**) and NK cells expanded by the combination of the αCD16 mAb with IrAPs (**C**). The assay was conducted in triplicate for each donor (*n* = 5). The statistical significance was determined by one-way ANOVA. ^#^ *P* < 0.05, ^##^ *P* < 0.005, ^###^ *P* < 0.0005 (^#^NK alone *versus* other groups). *P < 0.05, **P < 0.005, ***P < 0.0005 (*NK + IrAPs *versus* other groups).

We next evaluated phenotypic differences between resting and expanded NK cells. Resting and expanded NK cells were analyzed by flow cytometry, and then we compared the expression levels of CD3, CD56, CD16, NKG2D (CD314), NKp30 (CD337), NKp44 (CD336), NKp46 (CD335), 2B4 (CD244), and DNAM-1 (CD226) between resting and expanded NK cells (Fig. 3). As shown in Fig. 3, we observed significantly increased expression of the following activating receptors on NK cells expanded by a combination of the αCD16 mAb with IrAPs (as compared to resting NK cells): NKG2D, DNAM-1, 2B4, NKp30, NKp44, and NKp46. Nonetheless, CD3, CD56, and CD16 expression levels were not significantly changed. In addition, this expansion method produced significant differences in CD3, CD56, CD16, DNAM-1, 2B4, NKp30, NKp44, and NKp46 as compared to NK cells expanded by either the αCD16 mAb or IrAPs. NK cells expanded by either the αCD16 mAb or IrAPs showed significant differences in NKG2D, DNAM-1, 2B4/NKp46 (NK cells expanded by the αCD16 mAb), and NKp44 as compared to resting NK cells. In contrast, CD56 and CD16 expression levels significantly decreased, and NKp30 showed no significant change. Furthermore, there were significant differences in expression levels of DNAM-1, 2B4, NKp44, and NKp46 between NK cells expanded by the αCD16 mAb and IrAPs. In addition, NK cells expanded by the combination of the αCD16 mAb with IrAPs had negligible T-cell (CD3) contamination as compared to NK cells expanded by either the αCD16 mAb or IrAPs. T cells were hardly detectable during expansion (<1%). Thus, these results indicate that the combination of the αCD16 mAb with IrAPs for NK cell expansion may further upregulate NK cell-activating receptors.

### CD107a is highly expressed on NK cells expanded by the combination of the αCD16 mAb with IrAPs

**Figure 4:**
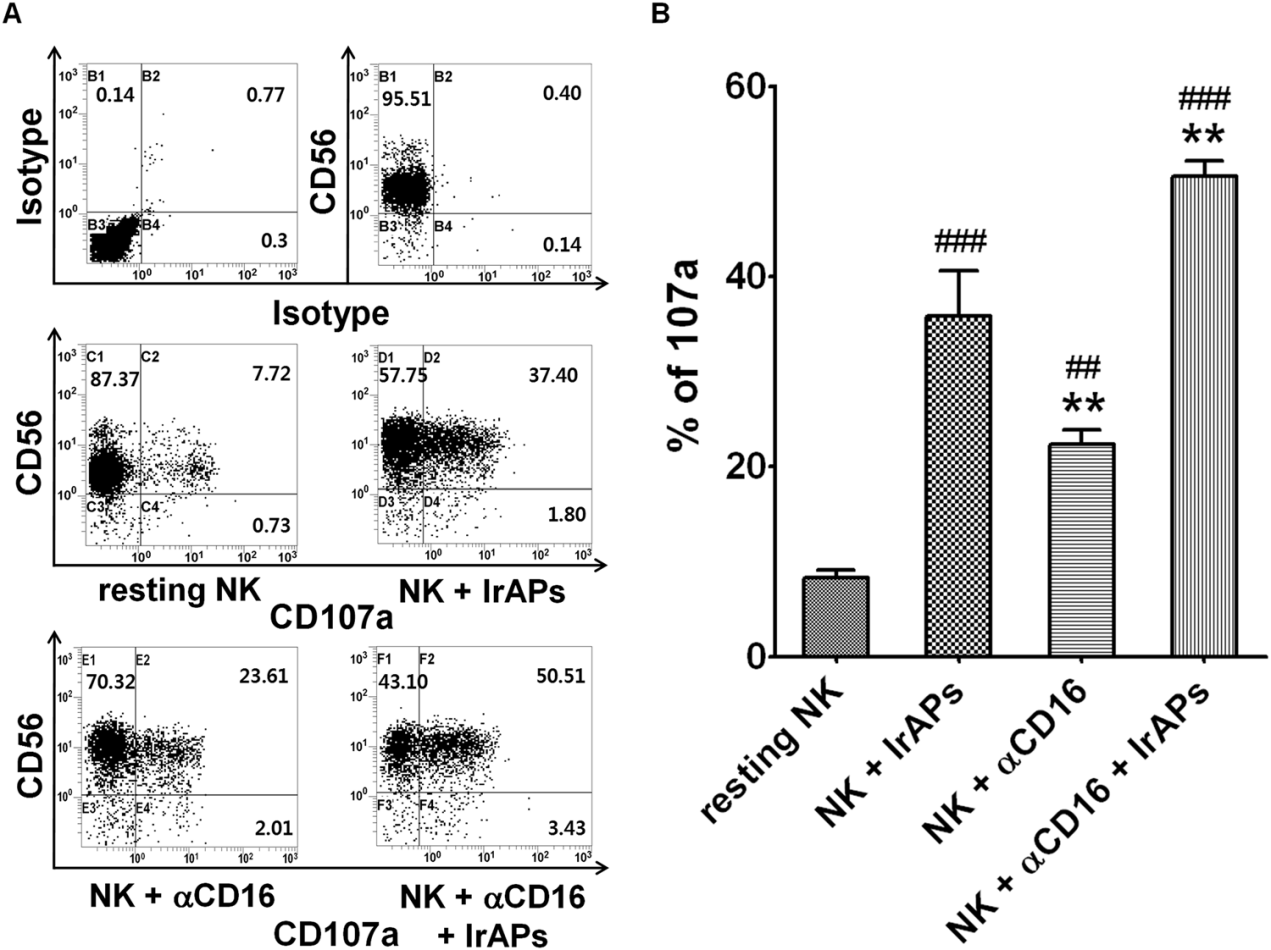
CD107a expression is upregulated in the NK cells expanded by the combination of the αCD16 mAb with IrAPs. CD107a analysis was performed as described in the Methods. (**A**) Representative FCM dot plots. (**B**) Comparison of expression levels of CD107a in NK cells expanded under various culture conditions. The assay was conducted in triplicate for each donor (*n* = 5). The statistical significance was determined by one-way ANOVA. ^##^ *P* < 0.005, ^###^ *P* < 0.0005 (^#^NK cells alone *versus* other groups). **P < 0.005, (*NK + IrAPs *versus* other groups).

CD107a expression correlates closely with activity of NK cells^30^. We determined whether degranulation marker CD107a was expressed on the surface of the NK cells expanded under various conditions. The expanded NK cells were incubated with K562 cells. After 4 h of incubation in the presence of monensin and an anti-CD107a mAb, NK cells were stained with anti-CD3 and anti-CD56 mAbs. As shown in Fig. 4, the resting NK cells expressed very little CD107a on the cell surface upon contact with K562 cells, but CD107a expression on the surface of NK cells (expanded under various culture conditions) increased more than 3.0-fold as compared to resting NK cells. In particular, CD107a expression on the surface of NK cells expanded by the combination of the αCD16 mAb with IrAPs increased 6.5-fold as compared to resting NK cells. Thus, these results indicate that NK cells expanded by the combination of the αCD16 mAb with IrAPs may further increase expression of CD107a caused by stimulation with target cancer cells.

### NK cells expanded by the combination of the αCD16 mAb with IrAPs show strongly increased secretion of IFN-γ after stimulation with target cancer cells

**Figure 5:**
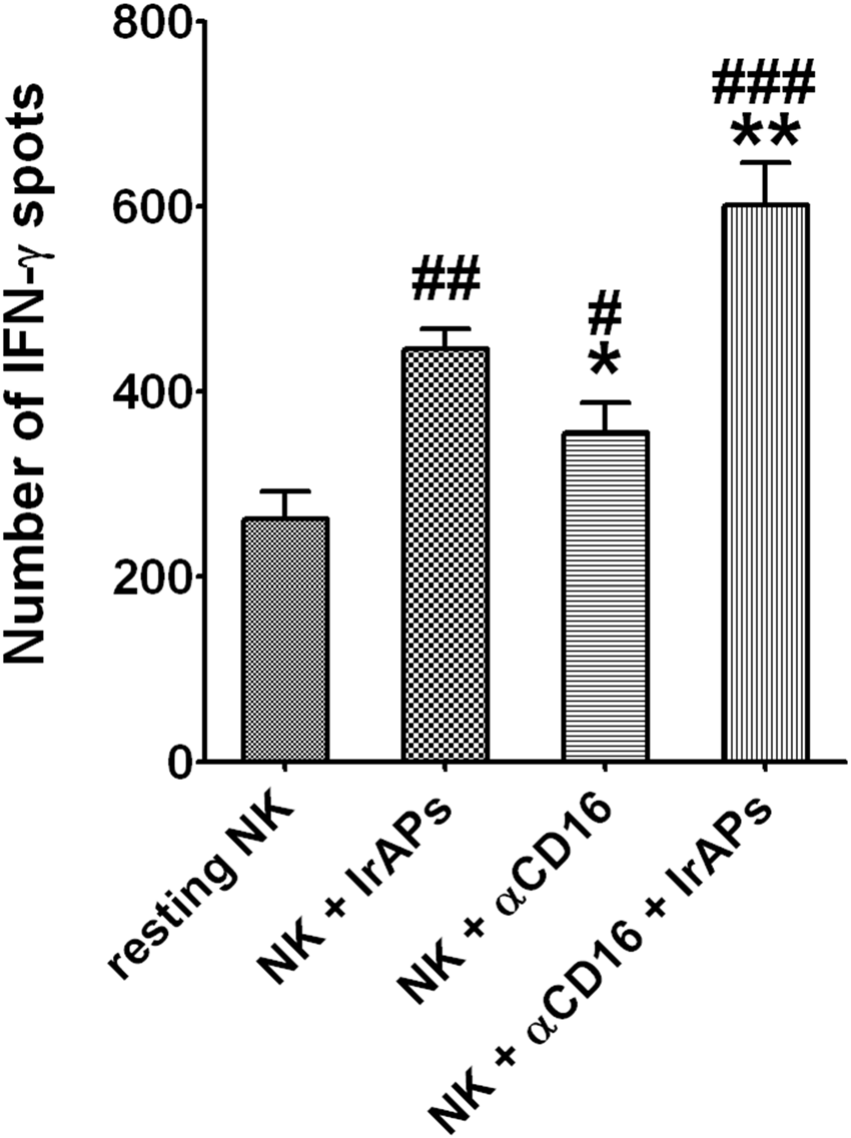
NK cells expanded by the combination of the αCD16 mAb with IrAPs show significantly increased secretion of IFN-γ after encountering target cancer cells. The IFN-γ ELISpot assays were performed as described in the methods. The results are shown as the average number of spots per 10^5^ responders ± SD. The assay was conducted in triplicate for each donor (*n* = 5). The statistical significance was determined by one-way ANOVA. ^#^ *P* < 0.05, ^##^ *P* < 0.005, ^###^ *P* < 0.0005 (^#^NK alone *versus* other groups). *P < 0.05, **P < 0.005, (*NK + IrAPs *versus* other groups).

We evaluated IFN-γ secretion of NK cells after stimulation with target cancer cells. An IFN-γ ELISpot assay was performed on resting and expanded NK cells. As shown in Fig. 5, resting NK cells secreted relatively low amounts of IFN-γ after stimulation with K562 cells, but NK cells expanded under various culture conditions strongly increased IFN-γ secretion. Specifically, NK cells expanded by the combination of the αCD16 mAb with IrAPs secreted larger amounts of IFN-γ than did NK cells expanded by either the αCD16 mAb or IrAPs. These results may be related to the CD107a expression. Thus, these findings indicate that NK cells expanded by the combination of the αCD16 mAb with IrAPs show a further increase in IFN-γ secretion after stimulation with target cancer cells.

### NK cells expanded by the combination of the αCD16 mAb with IrAPs show strongly improved antitumor cytotoxicity against target cancer cells

**Figure 6:**
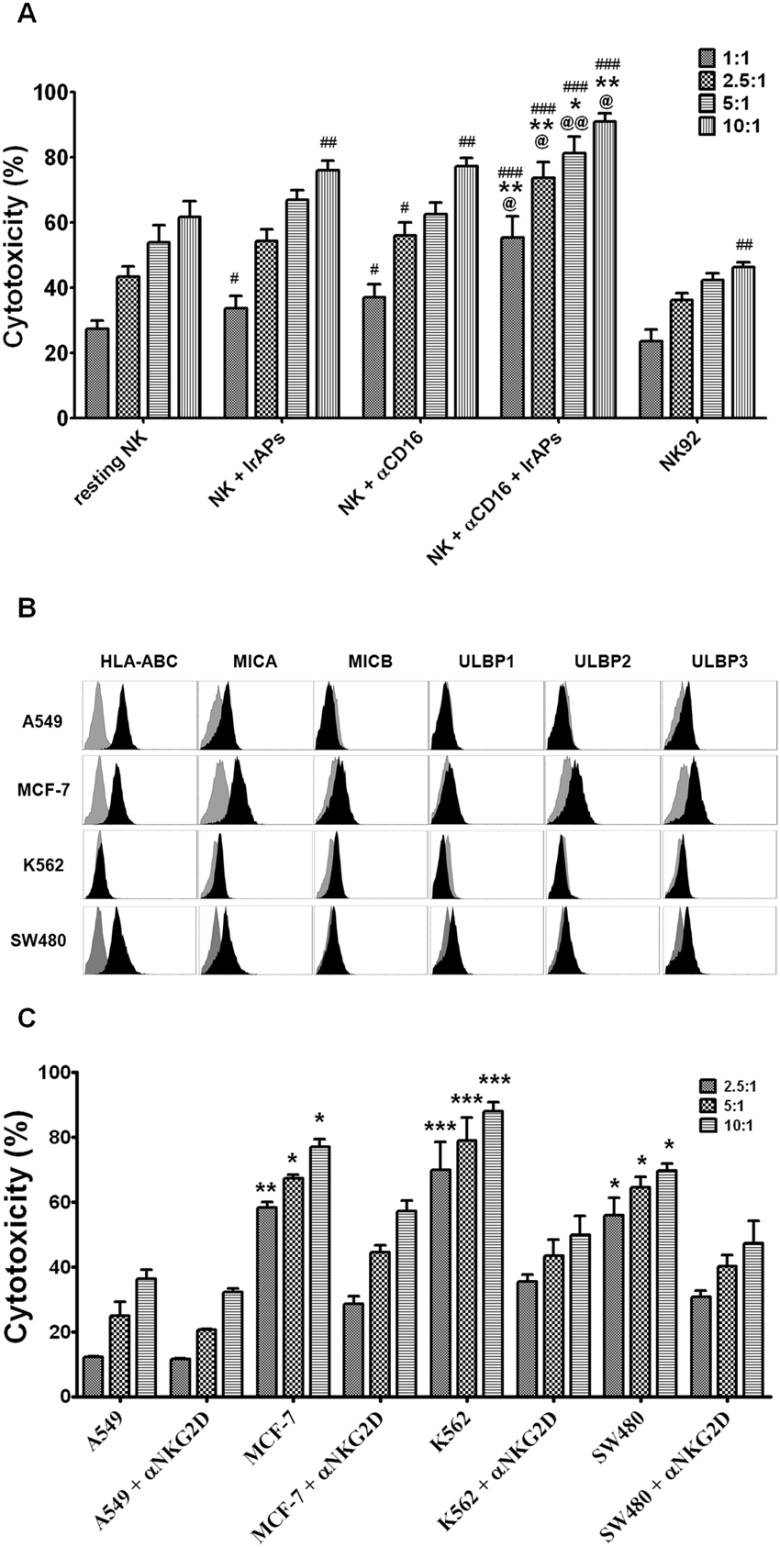
NK cells expanded by the combination of the αCD16 mAb with IrAPs show increased cytotoxic activity toward cancer cells. The cytotoxicity assays were performed as described in the methods. (**A**) Data are presented as the average cytotoxicity ± SD against K562 cells. (**B**) Data are presented as the average cytotoxicity ± SD against A549, MCF-7, K562, or SW480 cells. C, Representative FCM histograms for expression levels of MHC class I and NKG2D ligands on the various target cancer cells. Gray represents the isotype controls. All the experiments were conducted in triplicate for each donor (*n* = 5). The statistical significance was determined by one-way ANOVA. ^#^ *P* < 0.05, ^##^ *P* < 0.005, ^###^ *P* < 0.0005 (^#^NK alone *versus* other groups). *P < 0.05, **P < 0.005, ***P < 0.0005 (*NK + IrAPs *versus* other groups, target cancer cells *versus* NKG2D blocking). ^@^ *P* < 0.05, ^@@^ *P* < 0.005 (^@^NK + αCD16 *versus* NK + αCD16 + IrAPs).

We evaluated the antitumor cytotoxicity of expanded NK cells using an MHC class I-negative cell line (K562) and MHC class I-positive cell lines (MCF-7, A549, and SW480). As shown in Fig. 6A, antitumor cytotoxicity against target cancer cells was significantly elevated in the expanded NK cells compared to resting NK cells and NK-92 cells. In particular, NK cells expanded by the combination of the αCD16 mAb with IrAPs showed higher antitumor cytotoxicity than did NK cells expanded by either the αCD16 mAb or IrAPs. These results may be related to the CD107a expression and IFN-γ secretion. As shown in Fig. 6B, the most NK-sensitive target cancer cells, K562 cells, expressed NKG2D ligands but not MHC class I. A549 cells weakly expressed NKG2D ligands but MHC class I was strongly expressed. They were weakly sensitive to the NK cells expanded by the combination of the αCD16 mAb with IrAPs (Fig. 6C). Although MCF-7 and SW480 cells expressed MHC class I, these cells strongly expressed NKG2D ligands as compared to A549 cells. They were moderately sensitive to the NK cells expanded by the combination of the αCD16 mAb with IrAPs. NK-sensitive target cancer cells (K562, MCF-7, and SW480) tended to highly express NKG2D ligands or weakly express MHC class I as compared to NK-resistant target cells (A549). To evaluate the effect of NKG2D on the antitumor cytotoxicity of NK cells, NK cells expanded by the combination of the αCD16 mAb with IrAPs were cocultured with target cancer cells in the presence of a NKG2D-blocking antibody. Blocking of receptor NKG2D resulted in a substantial reduction in antitumor cytotoxicity against all target cancer cells except for A549 cells, which show low expression of NKG2D ligands (Fig. 6C). To investigate ADCC activity of expanded NK cells, SW480 cells were coated with cetuximab and co-incubated with expanded NK cells. Cetuximab-mediated ADCC activity was observed at various effector-to-target cell count ratios, and the highest ADCC activity was observed at an E:T ratio of 10:1 in each culture condition. In particular, NK cells expanded by the combination of the αCD16 mAb with IrAPs showed the highest cytotoxic activity in both cetuximab-treated and non-treated target cancer cells (Supplementary Fig. 1). Thus, these results indicate that NK cells expanded by the combination of the αCD16 mAb with IrAPs exert increased antitumor cytotoxicity against target cancer cells, and NKG2D is one of the important factors among NK cell-activating receptors. Furthermore, we demonstrated that expanded NK cells have the ability to more effectively kill target cancer cells by ADCC.

### NK cells expanded by the combination of the αCD16 mAb with IrAPs have strong antitumor effects against lung and colon cancer xenografts in NOD/SCID mice

**Figure 7:**
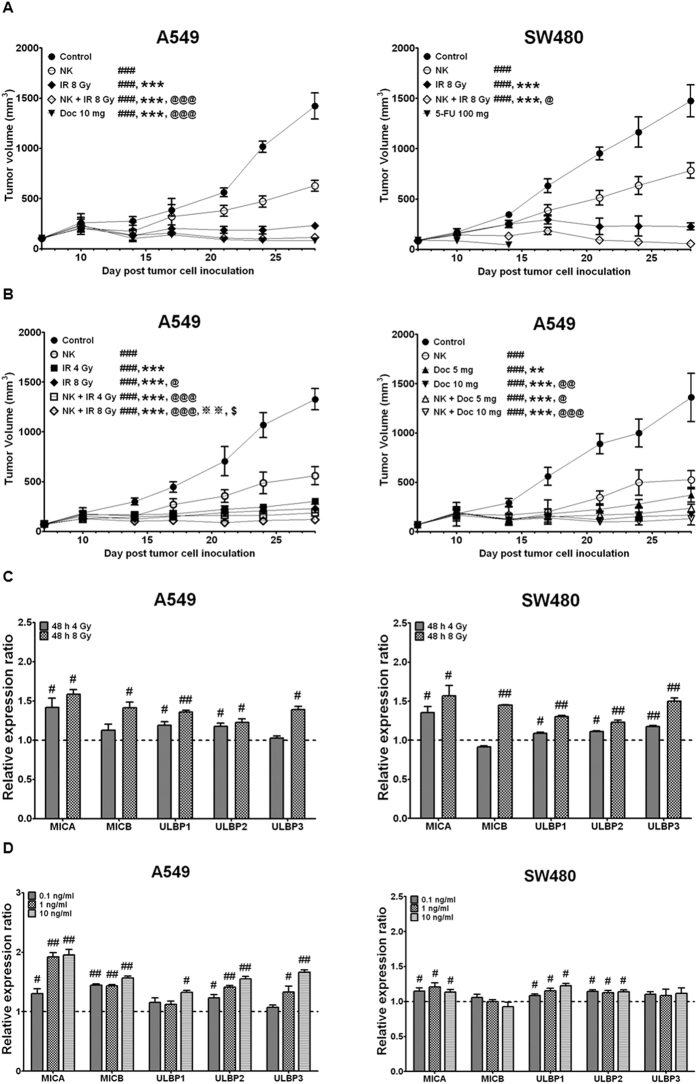
NK cells expanded by the combination of the αCD16 mAb with IrAPs have a strong *in vivo* antitumor effect in lung and colon cancer xenograft models. A549 cells (2 × 10^6^) and SW480 cells (5 × 10^6^) were subcutaneously injected into the right thigh of SCID mice. IR was applied at 4 and 8 Gy to the tumor in the right thigh of the mice. Expanded NK cells (10^7^) were intravenously injected into each mouse. 5-FU (100 mg/kg) or Doc (5 or 10 mg/kg) were administered into the tail vein 3 days before every NK injection. A and B, Tumor volume (length × width^2^ × 0.5) are expressed as the average tumor volume ± SD of five mice per group (A549- and SW480-bearing mice). (**C**,**D**) Relative NKG2D ligand expression ratios were calculated by dividing irradiated (**C**) and chemotherapeutic drug-treated (**D**) target cell MFI by untreated-target-cell MFI. All experiments performed independently three times. The statistical significance was determined by one-way ANOVA and paired Student’s *t* test. ^#^ *P* < 0.05, ^##^ *P* < 0.005, ^###^ *P* < 0.0005 (^#^Control *versus* other groups). ***P < 0.0005 (*NK *versus* other groups). ^@^ *P* < 0.05, ^@@^ *P* < 0.005, ^@@@^ *P* < 0.0005 (^@^IR at 8 Gy (Fig. 7A) *versus* other groups; IR at 4 Gy *versus* other groups; Doc 5 mg *versus* other groups). ^※※^ *P* < 0.005 (^※^IR at 8 Gy (Fig. 7B) *versus* NK + IR at 8 Gy). ^$^ *P* < 0.05, (^$^NK + IR at 4 Gy *versus* NK + IR at 8 Gy).

We evaluated the antitumor effect of the expanded NK cells against lung and colon cancer xenografts in NOD/SCID mice. SW480 human colon cancer cells and A549 human lung cancer cells were subcutaneously inoculated into the right thigh of NOD-SCID mice. Irradiation was applied at 4 or 8 Gy to the tumor in the right thigh of the mice. Then, the expanded NK cells were intravenously injected into each mouse. 5-Fluorouracil (5-FU) and docetaxel (Doc) were administered into the tail vein 3 days before every NK injection. 5-FU treatment, used as a reference drug, induced acute diarrhea after the first administration in most mice and eventually led to death. Therefore, more specific combination therapies were performed only in the A549 lung cancer model. As shown in Fig. 7, administration of expanded NK cells significantly inhibited tumor growth in both A549 lung cancer and SW480 colon cancer xenograft models. Of note, the antitumor efficacy of the expanded NK cells was further enhanced by the combined treatment with irradiation in both A549 lung cancer and SW480 colon cancer xenograft models (Fig. 7A and B). The 8-Gy irradiation was more effective at increasing the efficacy of the expanded NK cells as compared with the 4-Gy irradiation in A549 lung cancer xenograft models (Fig. 7B). Doc, used as a reference drug, strongly inhibited the growth of A549 lung cancer cells. The tumor volume in the mice treated with a 5 mg/kg dose of Doc combined with expanded NK cells was further significantly inhibited as compared with a single treatment, whereas the 10 mg/kg dose of Doc combined with expanded NK cells did not show significant differences from the Doc-alone group (10 mg/kg; Fig. 7B). Moreover, irradiation and chemotherapeutic agents significantly upregulated NKG2D ligand, which could the antitumor activity of NK cells (Fig. 7C,D). Taken together, these results revealed *in vivo* antitumor activity of the NK cells expanded by the combination of the αCD16 mAb with IrAPs in lung and colon cancer xenograft models. Furthermore, the combined treatment with irradiation or a chemotherapeutic drug further improved *in vivo* antitumor activity of the expanded NK cells.

## Discussion

In this study, we developed an improved method for large-scale expansion of NK cells using an anti-CD16 mAb and irradiated autologous PBMCs as feeder cells instead of cancer cell-based feeder cells. Feeder cells provide a suitable environment for the NK cell expansion through various mechanisms, including direct cell-to-cell interactions and production of soluble growth factors^31, 32^. CD16 (FcγRIII) is associated with the immunoreceptor tyrosine-based activation motif (ITAM)-containing FcεRI γ chain and CD3ξ chain^33^. Unlike other ITAM-coupled NK cell receptors, CD16 has the unique ability to activate resting human NK cells by itself, and activation by CD16 can be enhanced by other activation receptor signals^13^. Human NKG2D is associated with DAP10, which contains a tyrosine-based signaling motif (YINM)^34, 35^. Several studies have suggested that NKG2D stimulation induces strong activation of NK cells^36–39^. NKG2D is an important activating receptor and provides a coactivation signal to pre-existing other activation signals, such as CD16, NKp46, and 2B4^13, 40^. Our results also suggest that NKG2D is one of the key activation factors of NK cells in terms of antitumor cytotoxicity toward target cancer cells.

Ahn *et al*. reported that irradiated (20 Gy) and anti-CD3- and recombinant human IL-2 (rhIL-2)-stimulated PBMCs as feeder cells express larger amounts of ULBP1-3 as compared to fresh PBMCs, while MIC-A/B expression is not significantly altered^41^. We used the radiation dose of 25 Gy to inactivate lymphocytes in PBMCs. This dose of radiation effectivly blocks proliferation of T cells and increases the expression of various NKG2D ligands and CD48 on PBMCs. Some studies have shown that an irradiation dose of 25 Gy is optimal for effective inactivation of lymphocytes^42^.

2B4/CD48 interactions play a crucial role in the proliferation of NK cells^18, 43^. Although irradiated PBMCs expressed NKG2D ligands and CD48, which activates resting NK cells, additional activation signals are required for sufficient activation of resting NK cells. Unlike T cells, NK cells do not have a dominant activation receptor except for the ADCC induced by CD16. Thus, NK cell activation is regulated by combinations of synergistic receptors. Particularly, coengagement of CD16 with NKG2D or 2B4 further enhances the Ca^2+^ flux, cytokine production, and cytotoxicity toward cancer cells^13, 44^.

Nuclear factor κB (NF-κB) is a key transcription factor that induces the reprogramming of gene transcription for cytokine and chemokine production. NF-κB helps to elicit NK cell effector functions upon target cell recognition^45, 46^. NF-κB is regulated by multiple activating receptor-ligand interactions such as those involving NKG2D, 2B4, and DNAM-1, but single-receptor engagement is insufficient for NF-κB activation. Recently, it was reported that 2B4 and NKG2D (or DNAM-1) coactivation elicits synergistic NF-κB activation by inducing stepwise phosphorylation of p65^47^. Thus, combinations of different receptor signals may strongly induce NK cell activation, including natural cytotoxicity, cytokine production, and proliferation. Using blocking antibodies specific to each receptor, we demonstrated that coengagement of receptors NKG2D and 2B4 is essential for the activation and expansion of NK cells. In particular, the synergistic combination of CD16 with NKG2D and receptor 2B4 was found to be crucial for the optimal NK cell expansion.

In this study, irradiated autologous PBMCs alone were insufficient to effectively expand NK cells. Therefore, we developed a new *in vitro* expansion method using a combination of the anti-CD16 mAb (αCD16 mAb) with irradiated autologous PBMCs (IrAPs) for robust expansion of highly purified cytotoxic NK cells. This method expanded the NK cells more potently (>5000-fold). Moreover, the rate of increase of NK cell expansion is markedly higher than the sum of the increase rates of NK cell expansion by the αCD16 mAb and IrAPs separately. These findings indicate a synergistic effect of the αCD16 mAb and IrAPs on NK cell expansion. On day 21, the NK cells expanded by the αCD16 mAb and IrAPs were composed of highly purified CD3^−^CD56^+^ NK cells (>98%) with lesser T-cell contamination ( < 1%). They showed upregulation of NKG2D, NKp30, NKp44, NKp46, 2B4, DNAM-1, and CD107a and secreted larger amounts of IFN-γ after coculture with target cancer cells. The receptor balance of NK cells was changed by the combination of the αCD16 mAb with IrAPs toward activation and may result in enhancement of lysis of target cancer cells.

In this study, cytotoxic activity of expanded NK cells was triggered when the target cancer cells lack expression of MHC class I molecules and/or have higher expression of NKG2D ligands. Nevertheless, cytotoxicity was not completely inhibited by blocking of receptor NKG2D. DNAM-1, 2B4, NKp30, NKp44, and NKp46 or other unknown receptors may have affected cytotoxic activity in this case^13, 44, 48, 49^.

NK cells have two main effector functions: a direct cytotoxic effect and cytokine secretion. CD107a is known as a marker of degranulation of cytotoxic T cells or NK cells after stimulation^30, 50^. Alter *et al*. reported that CD107a expression correlates closely with NK cell functional activity such as cytokine secretion and target cell lysis^30^. Activated NK cells can secrete various effector cytokines such as IFN-γ and TNF-α. In particular, IFN-γ performs critical functions in antiviral defense, cancer surveillance, immunoregulation, and antitumor responses^51, 52^. Thus, the functional activity of NK cells expanded by the combination of the αCD16 mAb with IrAPs was demonstrated by means of degranulation marker CD107a, IFN-γ secretion, and antitumor cytotoxicity against target cancer cells.

We evaluated the antitumor effect of the expanded NK cells in the NOD/SCID mouse model of human lung and colon cancer. The expanded NK cells significantly inhibited tumor growth in both lung and colon cancer mouse models. Moreover, the antitumor effect of the expanded NK cells was further enhanced by combined treatment with irradiation. This phenomenon may be related to the upregulation of NKG2D ligand by irradiation. The latter can upregulate various immunologically important molecules that alter immunogenicity of cancer cells^53, 54^. Recently, it has been reported that irradiation can upregulate NKG2D ligand, which enhances the sensitivity of various cancer cells to NK cell-mediated cytotoxicity^55, 56^. The mice treated with a 5 mg/kg dose of Doc combined with expanded NK cells showed a significantly reduced tumor growth compared with a single group. Other reports and our experiments have shown that expression of NKG2D ligand on cancer cells can be increased by chemotherapeutic agents, resulting in enhanced NK cell-mediated cytotoxicity^56–59^. Unfortunately, most of the 5-FU-treated mice (SW480-positive control) died because of acute diarrhea after the first dose. This phenomenon may be due to the 5-FU properties and dose. The combination of Doc (10 mg/kg) with expanded NK cells did not significantly improve the antitumor effect as compared with Doc alone. Therefore, the dose and timing of administration of chemotherapeutic drugs should be carefully considered for combination with NK cells. Thus, we demonstrated *in vivo* antitumor activity of expanded NK cells in the lung and colon cancer xenograft models, and the combination treatment with irradiation or a chemotherapeutic drug further improved their antitumor effect.

Overall, cell-to-cell communication is crucial for coordination of cellular activation and proliferation. NK cells use combinations of synergistic receptors for activation and proliferation. We developed a new *in vitro* NK cell expansion method using a combination of the anti-CD16 mAb with irradiated autologous PBMCs without the use of cancer cells or other genetically modified feeder cells under GMP conditions. This method results in potent antitumor activity both *in vitro* and *in vivo*. Additionally, the combination of receptor signals such as CD16, NKG2D, and 2B4 could have a significant impact on the cascade of events leading to potent NK cell activation and proliferation and the control of cancer via immunity.

## Methods

### Human cancer cell lines

K562 (CCL-243), A549 (CCL-185), MCF-7 (HTB-22), and SW480 (CCL-288) cells were purchased from American Type Culture Collection (ATCC, Manassas, VA). Cells were cultured in a complete medium, which consisted of RPMI 1640 (K562, A549, SW480 cells) or DMEM (MCF-7 cells) supplemented with 10% of fetal bovine serum (FBS), 100 U/ml penicillin, and 100 μg/ml streptomycin, and were maintained at 37 °C in a humidified atmosphere containing 5% of CO_2_. Cell culture reagents were purchased from Life Technologies (Gaithersburg, MD).

### NK cell isolation and expansion

All experiments using human blood samples were approved by the Ethical Committee of Dongnam Institute of Radiological & Medical Sciences, and written informed consent was obtained from all the donors before enrollment. All methods were performed in accordance with the relevant guidelines and regulations. PBMCs were isolated from peripheral whole blood using Histopaque-1077 (Sigma-Aldrich, St. Louis, MO) density gradient centrifugation. For obtaining highly purified NK cells, non-NK cells were depleted by means of a magnetically activated cell sorting (MACS) system according to the manufacturer’s instructions (Miltenyi Biotec, Germany). NK cell purity was assessed by flow cytometry using anti-human CD3 and CD56 mAbs. Highly purified NK cells were seeded at 10^5^ cells/ml and cultured with or without irradiated (25 Gy) autologous PBMCs (10^6^ cells/ml) in 1 μg/ml anti-human CD16 mAb (clone B73.1; eBioscience, San Diego, CA)-coated culture plates. In addition, highly purified NK cells were cocultured at 10^5^ cells/ml with irradiated (5, 10, 15, 20, or 25 Gy) autologous PBMCs (10^6^ cells/ml) in uncoated culture plates. The culture medium was Lymphocyte Growth Medium 3 (LGM-3) (Lonza, USA) containing 500 IU/ml rhIL-2 (Proleukin, Switzerland) and 5% of human serum (Sigma-Aldrich). On day 5, expanded NK cells were transferred to an uncoated culture flask in the LGM-3 medium containing 500 IU/ml rhIL-2 and 5% of human serum. A fresh culture medium containing 500 IU/ml rhIL-2 and 5% of human serum was added to the flask every 2 to 3 days for 21 days. NK cell expansion procedures were performed under GMP conditions.

### Antibodies and flow cytometry

The phenotype of NK cells was assessed by flow cytometry on an FC 500 (Beckman Coulter, Fullerton, CA) with the following monoclonal antibodies against human proteins: anti-human CD3 phycoerythrin (PE)-conjugated antibody (clone UCHT1), anti-CD56 phycoerythrin-cyanine 5 (PE-Cy5)-conjugated (clone N901), anti-CD16 PE-conjugated (clone 3G8), NKG2D PE-conjugated (CD314; clone ON72), and HLA-ABC fluorescein isothiocyanate (FITC)-conjugated (clone B9.12.1) mAbs were purchased from Beckman Coulter. Anti-human NKp30 PE-conjugated (CD337; clone p30-15), NKp44 PE (CD336; clone p44-8.1), NKp46 PE (CD335; clone 9E2/Nkp46), DNAM-1 FITC (CD226; clone DX11), and 2B4 FITC (CD244; clone 2-69) mAbs were purchased from BD Pharmingen (San Diego, CA). Anti-human MICA PE-conjugated (clone 159227), MICB PE (clone 236511), ULBP-1 PE (clone 170818), ULBP-2 PE (clone 165903), and ULBP-3 PE-conjugated (clone 166510) mAbs were acquired from R&D Systems (Minneapolis, MN). The cells were also stained with their corresponding isotype control mAbs.

### The initial proliferation assay of NK cell

The cell proliferation was measured using the EZ-Cytox Cell viability assay kit (Daeillab Service, Seoul, Korea). For blocking of NKG2D and 2B4, NK cells were preincubated with or without 5 μg/ml anti-NKG2D (clone 149810; R&D systems) and/or 5 μg/ml anti-2B4 (clone C1.7; BioLegend) for 30 min at 4 °C. After that, 100 μl of NK cells (10^4^ cells/well) was added to each well of a 96-well plate with or without feeder cells (10^6^ cells/well). After 5 days, 10 μl of the EZ-Cytox solution was added to each well of the plate and incubated at 37 °C for 4 h. The absorbance was measured by means of the SoftMax Pro 4.3.1 software (Molecular Devices, Sunnyvale, CA) at 450 nm.

### Analysis of NK cell degranulation (CD107a)

NK cells were cocultured with target cells (K562) at a ratio of 1:1 in a final volume of 200 μl at 37 °C and 5% CO_2_ for 4 h in the presence of the FITC-conjugated anti-CD107a antibody (BD Biosciences) and Monensin (GolgiStop; BD Biosciences). After that, the cells were stained with PE-conjugated anti-CD3 and PE-Cy5-conjugated anti-CD56 antibodies for 30 min. The cells were then washed and analyzed by flow cytometry

### Analysis of IFN-γ secretion of NK cells by an enzyme-linked immunospot (ELISPOT) assay

The ELISPOT assay was performed per manufacturer’s instructions (Mabtech, Nacka, Sweden). Briefly, NK cells (10^5^) and target cancer cell line K562 (10^6^) were added into a flat-bottomed ELISPOT plate precoated with a capture antibody and containing 200 μl/well complete medium. The palate was incubated for 4 h at 37 °C in a humidified incubator with 5% of CO_2_. After washing with PBS, we added a detection antibody at 100 μl per well and incubated the plate for 2 h at room temperature. After a wash with PBS, we added a substrate solution at 100 μl per well and developed distinct spots. Finally, the spots were quantified using the AID ELISpot Reader System (Autoimmun Diagnostika GmbH, Germany).

### An NK cell-mediated cytotoxicity assay by flow cytometry

K562, A549, HCT116 and MCF-7 cells(target cells) were labeled with carboxyfluorescein succinimidyl ester (CFSE) at a final concentration of 5 μM for 15 min at 37 °C in a humidified incubator with 5% of CO_2_. After labeling, the cells were washed with a complete medium. NK cells (effector cells) were cocultured with CFSE-labeled K562 cells at the appropriate effector-to-target cell count ratios (10:1, 5:1, 2.5:1, 1:1) in round-bottomed 96-well plates at 37 °C in a humidified incubator with 5% of CO_2_ for 4 h. For blocking NKG2D, NK cells were preincubated with 5 μg/ml anti-NKG2D antibody (clone 149810; R&D Systems) for 30 min at 4 °C. After that, the cells were transferred to tubes and placed in an ice-water bath. NK cells (effector cells) were cocultured with CFSE-labeled target cells at the appropriate effector-to-target cell count ratios (10:1, 5:1, or 2.5:1). Propidium iodide PI (Sigma-Aldrich, St. Louis, MO) at 50 μg/ml was added for labeling of DNA of dead cells. Dead cells were analyzed by flow cytometry.

### NKG2D ligand expression under the influence of irradiation or a chemotherapeutic drug

A549 and SW480 cells were cultured for 24 h in a complete medium, which consisted of RPMI 1640 supplemented with 10% of FBS, 100 U/ml penicillin, and 100 μg/ml streptomycin, and were maintained at 37 °C in a humidified atmosphere containing 5% of CO_2_. The cells were irradiated (4 or 8 Gy), treated with Doc (0.1, 1, or 10 ng/ml) or 5-FU (0.1, 1, or 10 ng/ml). After 2 days, the cells were stained for MICA, MICB, ULBP1, ULBP2, and ULBP3 for 30 min. The cells were analyzed by flow cytometry.

### The xenograft model

Five-week-old nonobese diabetic (NOD) severe combined immunodeficiency (SCID) NOD.CB17-Prkdcscid/ARC (NOD-SCID) mice were purchased from Central Lab, Animal Inc. (Seoul, Korea). Experiments on the animals were approved by the Dongnam Institute of Radiological and Medical Sciences Institutional Animal Care and Use Committee. All methods were performed in accordance with the relevant guidelines and regulations. SW480 human colon cancer cells (5 × 10^6^) and A549 human lung cancer cells (2 × 10^6^) were subcutaneously inoculated into the right thigh of NOD.CB17-Prkdcscid/ARC mice. When the tumor grew to approximately 50–100 mm^3^ in volume, irradiation was applied at 4 or 8 Gy to the tumor in the right thigh of mice using a linear accelerator (Infinity; Elekta, UK). After 2–3 h, NK cells (10^7^) were administered into the tail vein. Tumor volume (length × width^2^ × 0.5) was measured twice a week. Irradiation and NK cell injection were performed three times at 1-week intervals. 5-FU (100 mg/kg, SW480 positive control) and Doc (10 mg/kg, A549 positive control) were administered into the tail vein 3 days before every NK injection.

### Statistical analysis

This analysis was performed using one-way analysis of variance (ANOVA) and paired Student’s *t* test. Differences were considered statistically significant at *P < *0.05.

### Data availability

All relevant data are available from the corresponding author Y.-S.P. or K.Y. on request.

## Electronic supplementary material

Supplementary Information
