# Supplementary material for: Expansion of cytotoxic natural killer cells using irradiated autologous peripheral blood mononuclear cells and anti-CD16 antibody
Source: Sci Rep. 2017 Sep 11;7:11075. doi: 10.1038/s41598-017-09259-1 (PMC5593981; doi:10.1038/s41598-017-09259-1)
Supplement: Supplementary file 1 — Supplementary Information [file 41598_2017_9259_MOESM1_ESM.doc]

**Supplementary information**

**Expansion of cytotoxic natural killer cells using irradiated autologous peripheral blood mononuclear cells and anti-CD16 antibody**

Hong-Rae Lee^1,2,‡^, Cheol-Hun Son^1,‡^, Eun-Kyoung Koh^1^, Jae-Ho Bae^2^, Chi-Dug Kang^2^, Kwangmo Yang^1^,* & You-Soo Park^1^,*

^1^Department of Research Center, Dongnam Institute of Radiological & Medical Sciences, Jwadong-gil 40, Jangan-eup, Gijang-gun, Busan 46033, South Korea

^2^Department of Biochemistry, Pusan National University School of Medicine, Yangsan 50612, South Korea

***Corresponding author:** Correspondence and requests for materials should be addressed to Y.-S.P. (email: biotek01@hanmail.net) or K.Y. (email: kmyang@dirams.re.kr). All subsequent correspondence should be directed to Y.-S.P.

^‡^These authors contributed equally to this work.

**Supplementary Figures and Legends**

**Supplementary Fig. 1. NK cell-mediated antibody-dependent cellular cytotoxicity (ADCC) in target cancer cells.** SW480 cells were coated with cetuximab and co-incubated with NK cells expanded under various culture conditions. ADCC activity was analyzed by flow cytometry. Data are presented as the average cytotoxicity ± SD against target cancer cells. The assay was conducted in triplicate for each donor (*n*=5). The statistical significance was determined using paired Student’s *t* test. **P* < 0.05, ***P* < 0.005, ****P* < 0.0005 (*NK cells + SW480 *versus* NK cells + cetuximab-treated SW480).

**Supplementary Methods**

**ADCC assay.** To evaluate ADCC, SW480 cells were labeled with carboxyfluorescein succinimidyl ester (CFSE) at a final concentration of 5 µM for 15 min at 37˚C in a 5% CO_2_ incubator. After labeling, the cells were washed with a complete medium. NK cells (effector cells) were cocultured with CFSE-labeled SW480 cells at the appropriate effector-to-target cell count ratios (10:1, 5:1, 2.5) in round-bottomed 96-well plates at 37˚C in a 5% CO_2_ incubator for 4 h. Cetuximab (Merck Millipore, Germany) was incubated with CFSE-labeled SW480 cells at concentration of 10 µg/ml in a 5% CO_2_ incubator for 1 h before addition of effector cells. Propidium iodide PI (Sigma-Aldrich, St. Louis, MO) at 50 µg/ml was added for labeling of DNA of dead cells. Dead cells were analyzed by flow cytometry.
